# *Amanita phalloides* Mushroom Poisonings — Northern California, December 2016

**DOI:** 10.15585/mmwr.mm6621a1

**Published:** 2017-06-02

**Authors:** Kathy T. Vo, Martha E. Montgomery, S. Todd Mitchell, Pieter H. Scheerlinck, Daniel K. Colby, Kathryn H. Meier, Susan Kim-Katz, Ilene B. Anderson, Steven R. Offerman, Kent R. Olson, Craig G. Smollin

**Affiliations:** ^1^Department of Emergency Medicine, University of California, San Francisco; ^2^California Poison Control System, San Francisco Division; ^3^Department of Emergency Medicine, Alameda County Medical Center/Highland Hospital, Oakland, California; ^4^Department of Family Medicine, Dignity Health Dominican Hospital, Santa Cruz, California, ^5^Department of Emergency Medicine, University of California, Davis; ^6^California Poison Control System, Sacramento Division; ^7^Department of Clinical Pharmacy, University of California, San Francisco; ^8^Medical Toxicology Consultation Service, Kaiser Permanente Northern California.

*Amanita phalloides*, colloquially known as the “death cap,” belongs to the Phalloideae section of the Amanita family of mushrooms and is responsible for most deaths following ingestion of foraged mushrooms worldwide (*1*). On November 28, 2016, members of the Bay Area Mycological Society notified personnel at the California Poison Control System (CPCS) of an unusually large *A. phalloides* bloom in the greater San Francisco Bay Area, coincident with the abundant rainfall and recent warm weather. Five days later, CPCS received notification of the first human *A. phalloides* poisoning of the season. Over the following 2 weeks, CPCS was notified of an additional 13 cases of hepatotoxicity resulting from *A. phalloides* ingestion. In the past few years before this outbreak, CPCS received reports of only a few mushroom poisoning cases per year. A summary of 14 reported cases is presented here. Data extracted from patient medical charts revealed a pattern of delayed gastrointestinal manifestations of intoxication leading to dehydration and hepatotoxicity. Three patients received liver transplants and all but one recovered completely. The morbidity and potential lethality associated with *A. phalloides* ingestion are serious public health concerns and warrant medical provider education and dissemination of information cautioning against consuming foraged wild mushrooms.

**Initial case**. A man aged 37 years (patient A) picked two wild mushrooms in Santa Rosa, California (Table). He cooked and ate one mushroom, and approximately 10 hours later developed nausea, vomiting, and diarrhea. He was evaluated in a local emergency department (ED) for abdominal discomfort 20.5 hours after ingestion. A mycologist identified the uncooked mushroom sample provided by the patient as *A. phalloides*. Initial laboratory findings were notable for an elevated white blood cell count with lactate elevation and elevated creatinine suggesting dehydration (Table). Liver function tests (LFTs) 6 hours later showed elevated aspartate aminotransferase (AST) (92 IU/L; normal = 15–41) and alanine aminotransferase (ALT) (95 IU/L; normal = 17–63) levels. He was treated with aggressive intravenous (IV) fluid hydration, IV octreotide,* and IV silibinin.^†^ Two days after ingestion, the patient’s LFTs peaked at AST = 6,159 IU/L, ALT = 3,084 IU/L, total bilirubin = 2.9 mg/dL (normal = 0.2–1.2), and international normalized ratio (INR) (standardized prothrombin time) 3.2 units (normal = 0.8–1.2). Gastrointestinal symptoms and laboratory values gradually improved, and the patient was discharged home on day 6.

**TABLE:** Demographic and clinical data for 14 patients reported to the California Poison Control System after *Amanita phalloides* ingestion — Northern California, December 2016

Patient	Age (yr.)	Sex	Mushrooms ingested	Ingestion to symptom onset (hrs)	Ingestion to hospital admission (hrs)	Initial lactate (mmol/L)*	Initial BUN/Cr^†^ (mg/dL)	Initial AST/ALT^§^ (U/L)	Peak AST/ALT, INR (units)^¶^	Hospitalization duration (days)	Outcome
A	37	M	1 (stalk and cap)	10	20	5.2	27/1.4	31/40	3084/6159, 3.2	6	Recovered
B**	26	F	4 caps	9	20	5.87	21/0.64	51/45	11427/9693, 3.2	6	Recovered
C**	28	M	3 caps	9	23	2.98	20/1.46	444/454	6123/4401, 1.4	6	Recovered
D**	1.5 (18 mo.)	F	½ cap	9	19	9.22	16/<0.38	70/47	14300/10200, 10.2	36	Liver transplant, permanent neurologic impairment
E**^,††^	38	F	1 (stalk and cap)	9	48	7.0	24/0.8	1712/1025	9573/6239, 13.3	13	Liver transplant, recovered
F**	49	F	“Pieces”	9	48	6.72	95/2.24	1038/1100	11940/11350, 4.5	6	Recovered
G	36	M	½ cap	7	12	1.5	18/0.6	32/29	1858/3526, 1.6	5	Recovered
H	56	M	Multiple 8–10 cm caps	12	64	5.4	62/2.33	1599/3200	2820/5599, >13.3	16	Liver transplant, recovered
I	86	F	Unknown	Unknown	~48	Not drawn	64/1.11	768/1084	768/1084, 1.7	3	Recovered
J	93	F	Unknown	Unknown	~48	1.4	64/2.74	765/672	1497/1994, 1.8	9	Recovered
K	19	M	4 caps	12	29	Not drawn	18/0.92	89/151	113/184, 1.2	5	Recovered
L	19	M	8 caps	9	21	1.7	23/1.95	27/29	1404/2544, 2.1	5	Recovered
M^††^	22	M	2 “shots” of mushroom juice and 3 (stalk and cap)	<12	64	3.2	24/1.31	887/1326	2044/3351, 5.2	9	Recovered
N	22	M	1 “shot” of mushroom juice	4	64	1.7	18/0.94	6344/6400	6344/6400, 2.5	6	Recovered

**Abbreviations:** ALT = alanine transaminase; AST = aspartate transaminase; BUN = blood urea nitrogen; Cr = creatinine; F = female; INR = international normalized ratio; M = male.

* Normal lactate = 0.5–2.2 mmol/L.

^†^ Normal BUN = 7–20 mg/dL; normal Cr = 0.8–1.2 mg/dL.

^§^ Normal AST = 15–41 U/L; normal ALT = 17–63 U/L.

^¶^ Normal INR = 0.8–1.2 units.

** Part of a single household cluster of five patients.

^††^ Discharged from initial hospital with diagnosis of gastroenteritis.

**Household cluster.** A woman aged 26 years (patient B) prepared and grilled wild mushrooms for dinner for her husband (patient C, 28 years), her daughter (patient D, 18 months), her sister (patient E, 38 years), and a female friend (patient F, 49 years). The mushrooms had been given to her by a person she did not know, who reportedly picked them earlier in the day in the mountains. The mother, father, and child ate four, three, and one-half mushroom caps, respectively; the mother’s sister ate one cap and stalk, and the friend ate “pieces.”

All persons who consumed the mushrooms developed nausea, vomiting, and diarrhea approximately 9 hours after ingestion. The mother, father and child (patients B, C, and D) visited the ED 20 hours after ingestion with dehydration and gastrointestinal distress. All patients had laboratory values consistent with hepatic injury (Table). The patients were treated with aggressive IV fluid hydration, IV octreotide, and IV silibinin. Two days after ingestion of the mushrooms, the mother’s LFTs peaked at AST 11,427, ALT 9,693, total bilirubin 2.4 mg/dL, and INR 3.2 units. The father’s LFTs peaked on hospital day 1 with AST 6,123 IU/L, ALT 4,401 IU/L, and total bilirubin 2.8 on hospital day 2. Both parents’ symptoms improved, and they were discharged on hospital days 6 and 4, respectively. The child developed irreversible fulminant hepatic failure and required mechanical ventilation because of hepatic encephalopathy. She underwent a liver transplant 6 days after ingestion of the mushroom with a complicated postoperative course that included cerebral edema and permanent neurologic impairment.

The sister of the woman who prepared the meal (patient E) visited the ED before her other family members, but was discharged home after administration of IV fluids and antiemetic medications, with a diagnosis of gastroenteritis. She returned to the ED the following day with persistent nausea, vomiting, diarrhea, and abdominal cramping. At that time, her AST was 1,712 IU/L, ALT 1,025 IU/L, total bilirubin 2.0 mg/dL, and INR 1.8 units. She was treated with aggressive IV fluid hydration, IV octreotide, and IV silibinin, as well as the placement of a biliary drain, but developed irreversible fulminant hepatic failure and underwent liver transplant on hospital day 4, with subsequent improvement of her hepatic function.

The family friend (patient F) visited an ED 2 days after ingestion of the mushrooms, complaining of abdominal pain, nausea, vomiting, and diarrhea. On hospital day 1, her LFTs peaked at AST 11,940, ALT 11,350, and INR 4.5. She was treated with aggressive IV fluid hydration, IV octreotide, and IV silibinin. Her hepatic function recovered, and she was discharged home on hospital day 6.

**Additional cases.** In the subsequent 2 weeks, CPCS was notified of eight additional cases of acute liver injury after consumption of wild mushrooms in northern California counties (Table). One case involved a man aged 36 years who had ingested mushrooms, later confirmed to be *A. phalloides*, obtained from a friend who picked them during a hike. Another case occurred in a man aged 56 years who was evaluated at an ED 2 days after ingestion of foraged mushrooms and required a liver transplant; two cases occurred in women aged 86 and 93 years who received wild-picked mushrooms from a friend; and four men aged 19–22 years who developed hepatotoxicity after ingesting what they thought were psychedelic mushrooms picked from the wild. Most of these patients had recovery of hepatic function.

## Discussion

Over the course of 2 weeks in December 2016, CPCS investigated 14 suspected *A. phalloides* ingestions in five northern California counties. Eleven patients recovered, although three required liver transplants because of irreversible fulminant hepatic failure. One of those patients, a child, developed cerebral edema and suffered permanent neurologic sequelae.

Amatoxins, consisting of alpha, beta, and gamma amanitin, account for >90% of deaths related to mushroom poisoning worldwide (*1*). *A. phalloides* contains the alpha variety of amanitin, a cyclic octapeptide thought to be the primary agent of toxicity in humans (*2*). The amanitins are heat stable and are not inactivated by cooking. Once ingested, amatoxin is readily absorbed from the gastrointestinal tract into the portal circulation where it is taken up by hepatocytes, binding to DNA-dependent RNA polymerase (II) and halting intracellular protein synthesis, ultimately resulting in cell death (*3*). A lethal dose can be as low as 0.1 mg/kg, and a single mushroom can contain up to 15 mg (*1*). The clinical course of amatoxin poisoning is described in three phases: delayed gastroenteritis with significant body fluid volume loss (after a postingestion latency of 6–24 hours), symptomatic recovery (24–36 hours after ingestion), and fulminant hepatic and multiorgan failure (typically 3–5 days after the ingestion) (*4*). Patients who are evaluated early in the course of their illness might be discharged home only to return later with indications of liver failure, contributing to the relatively high case fatality rate (10%–20%) (*5*,*6*). Initial treatment emphasizes early supportive care including aggressive fluid and electrolyte replacement. In the event of irreversible fulminant liver failure, liver transplant might be required. A variety of therapies including multidose activated charcoal, high-dose penicillin, N-acetylcysteine, cimetidine, biliary drainage, and octreotide have been attempted with no definitive evidence of efficacy. Uncontrolled observational studies of *Amanita* intoxication suggest that the early use of silibinin, a milk thistle derivative, is associated with a reduction in mortality when compared with historical controls (*7*); however, as with the other aforementioned therapies, evidence supporting efficacy is lacking because of difficulties associated with conducting randomized controlled trials. Intravenous silibinin is licensed in Europe, and a clinical trial to evaluate its efficacy in treatment of hepatic failure induced by *Amanita* mushroom poisoning is currently underway in the United States.^§^ The majority of silibinin-treated patients in this report received the drug as participants of this trial.^¶^ Medical providers should contact the regional poison control center or a medical toxicology consultant to assist in the management of any patient with suspected amatoxic mushroom ingestion.

In California, *A. phalloides* species grow in a symbiotic relationship with coast live oak and other hardwood trees (*8*). They can be especially abundant in the early wet winter months, though the foggy coastal climate and warmer temperatures can support mushroom growth throughout the year (*4*). In 2016, local mycologists noted an abundance of wild mushroom growth, and California county health departments reported an increase in the incidence of mushroom poisoning (*9*). Although weather conditions and increased numbers of *A. phalloides* poisonings do not prove a cause and effect relationship, early seasonal rainfall and warmer subsequent temperatures made a substantial contribution to mushroom proliferation. In addition, a general increase in naïve foraging and wildcrafting (i.e., gathering plant material from its native environment for food or medicinal purposes) activities raises risk for poisoning.

Mycologists recommend exercising caution when foraging or purchasing wild mushrooms for consumption. If wild mushrooms are to be consumed, specimens should first be examined, identified, and deemed edible by an experienced mycologist (*4*). Prompt identification of mushroom-related toxic symptoms in the ED and early, aggressive IV volume replacement are critical first steps in diminishing the significant morbidity and mortality associated with amatoxin ingestion. Antidotal therapies might also be considered in conjunction with a consultant experienced in hepatotoxic mushroom poisoning. In patients with severe poisoning, early contact with the nearest liver transplant center is recommended. Response to this outbreak included the notification of the local counties and state department of public health, which subsequently issued a widely distributed press release (*9*). Measures to disseminate information regarding the dangers of *A. phalloides* ingestion are ongoing.

**Summary**

**What is already known about this topic?**

Ingestion of *Amanita phalloides* is responsible for a majority of mushroom-related deaths worldwide. Amatoxins, the principal toxic alkaloids found in these fungi, cause cell injury by halting protein synthesis. A possible antidote licensed in most of Europe, intravenous silibinin, is undergoing evaluation by clinical trial in the United States.

**What is added by this report?**

In December 2016, fourteen cases of *Amanita phalloides* poisoning were identified by the California Poison Control System (CPCS) among persons who had consumed foraged wild mushrooms. In the past few years before this outbreak, CPCS only received reports of a few mushroom poisoning cases per year. All patients in this outbreak had gastrointestinal manifestations of intoxication leading to dehydration and hepatotoxicity. Three patients received liver transplants; all patients recovered, although one (a child) had permanent neurologic impairment.

**What are the implications for public health practice?**

Wild-picked mushrooms should be evaluated by a trained mycologist before ingestion. Inexperienced foragers should be strongly discouraged from eating any wild mushrooms. Health care providers should be aware of the potential for toxicity after wild mushroom ingestion, that gastrointestinal symptoms mimicking viral gastroenteritis can occur after ingestion and slowly progress to potentially fatal hepatotoxicity, and should contact the local poison center for reporting and assistance with management of these patients.

## Footnotes

* Octreotide, by preventing emptying of the gallbladder, might reduce recirculation of amatoxins in bile to the liver.

† Silibinin dihemisuccinate, a milk thistle extract, competitively inhibits hepatic amatoxin uptake and enterohepatic recycling, and is available in the United States through an open clinical trial.

§ Intravenous Milk Thistle. (Silibinin-Legalon®SIL) for Hepatic Failure Induced by Amatoxin/Amanita Mushroom Poisoning. https://clinicaltrials.gov/ct2/show/NCT00915681.

¶ Registration identifier NCT00915681. https://clinicaltrials.gov/ct2/show/NCT00915681.
